# Susceptibility Profiles of* Mycobacterium ulcerans* Isolates to Streptomycin and Rifampicin in Two Districts of the Eastern Region of Ghana

**DOI:** 10.1155/2016/8304524

**Published:** 2016-12-14

**Authors:** Enid Owusu, Mercy Jemima Newman, Nana Konama Kotey, Amos Akumwena, Elizabeth Bannerman

**Affiliations:** ^1^Department of Medical Laboratory Sciences, School of Biomedical and Allied Health Sciences, University of Ghana, Accra, Ghana; ^2^Department of Medical Microbiology, School of Biomedical and Allied Health Sciences, University of Ghana, Accra, Ghana; ^3^Paakro Health Centre, Ghana Health Service, Paakro, Ghana

## Abstract

*Background*. Drug resistance is a major challenge in antibiotic chemotherapy. Assessing resistance profiles of pathogens constitutes an essential surveillance tool in the epidemiology and control of infectious diseases, including Buruli ulcer (BU) disease. With the successful definitive management of BU using rifampicin and streptomycin, little attention had been paid to monitoring emergence of resistant* Mycobacterium ulcerans (M. ulcerans)* isolates in endemic communities. This study investigated the susceptibility profiles of* M. ulcerans* isolates from two BU endemic areas in Ghana to streptomycin and rifampicin.* Methods*. The antibiotic susceptibility of seventy (70)* M. ulcerans* isolates to rifampicin and streptomycin was determined simultaneously at critical concentrations of 40 *µ*g/mL and 4 *µ*g/mL, respectively, by the Canetti proportion method.* Results*. Resistance to rifampicin was observed for 12 (17.1%)* M. ulcerans* isolates tested, whilst 2 (2.9%) showed resistance to streptomycin. None of the isolates tested showed dual resistance to both rifampicin and streptomycin.* Conclusion*. Outcomes from this study may not be reflective of all BU endemic communities; it, however, provides information on the resistance status of the isolates, which is useful for monitoring of* M. ulcerans*, as well as BU disease surveillance and control.

## 1. Introduction

Buruli ulcer (BU) disease, a chronic, necrotic mycobacteriosis of the skin, is characterised by lesions including nodules, papules, plaques, oedema, and large necrotic ulcers [1, 2]. It causes morbidity in affected persons with accompanying ominous socioeconomic consequences for both patients and their dependents [3]. The disease, which is caused by* Mycobacterium ulcerans*, has been reported in over thirty tropical and subtropical countries globally, including Ghana [4, 5]. Previously, treatment of BU disease depended on surgical excision coupled with plastic surgery and skin transplantation [6]. Currently, the administration of rifampicin and streptomycin for an eight-week period is being used successfully [7–9]. This was based on a recommendation by the World Health Organization (WHO), after a successful clinical trial by Etuaful et al., in Agroyesum in Ghana [10, 11]. Despite this success, new, recurrent, and unresponsive cases of Buruli ulcer are reported from endemic areas [12–14]. These challenges, coupled with the misuse, abuse of recommended doses of antibiotics, and noncompliance on the part of patients have the potential to generate mutant strains with resistant properties, as has been reported for other cases of mycobacteriosis particularly tuberculosis (TB) [14, 15].* Mycobacterium ulcerans* mutation, though reportedly low (0.9%) even for rifampicin, exists and has the potential to undermine instituted interventions for the control of the disease [14].

Currently, there is little information regarding the status of resistant* M. ulcerans* strains circulating within endemic communities. The reportedly low mutation rate is also presumably the reason for the complacent inertia associated with few studies reporting on the resistance to streptomycin and rifampicin since the recommendation by the WHO [11, 13]. Effective control measures however require disease monitoring with effective public health tools.

Periodic investigation of the susceptibility of microbial isolates including* M. ulcerans* to established drug regimen by laboratory methods is one such important public health monitoring tool. Monitoring* M. ulcerans* isolates circulating in BU endemic areas is therefore crucial to effective disease control [12]. This will help assess local rates of circulating resistant* M. ulcerans* isolates as this is critical to effective disease control. Information obtained will be crucial to policy formulation on disease and patients and review and implementation as well as optimizing drug therapy particularly in BU endemic areas. Therefore, this study reports on the susceptibility profiles of* M. ulcerans* isolates obtained from Paakro in the Akuapem South district and Asuboi in the Suhum-Kraboa-Coaltar district of the Eastern region, two largely understudied BU endemic areas in Ghana.

## 2. Methods

This was a prospectively designed experimental study, where data was collected after obtaining ethical clearance from the Ethical Review Boards of Ghana Health Service (GHS) and the Noguchi Memorial Institute for Medical Research (NMIMR) (Study number 040/09-10).

### 2.1. Study Site, Sample Collection, and Preparation

Over a two-year period, June 2010 to April 2012, a total of 540 swab specimens were obtained from 135 clinically confirmed BU patients reporting to the two health centres of Paakro and Asuboi with treatment failures and chronic wounds lasting longer than expected. These swab specimens had exudates from lesions of the BU cases and were used for laboratory confirmation of the presence of* M. ulcerans*. Exudates were cultured on Lowenstein-Jensen (L-J) for 8–12 weeks. Growth of typical* M. ulcerans* colonies were detected on seventy of the cultures from 70 different patients, after an incubation period spanning eight to twelve weeks at 32°C on L-J [16].* M. ulcerans* colonies were confirmed by conventional laboratory methods [17] by subjecting the cultures with growth to Ziehl-Neelsen staining and a confirmatory IS2404 PCR [17]. Briefly, for the Ziehl-Neelsen staining procedure, smears were prepared from the cultures with growth and allowed to dry, after which they were heat-fixed and flooded with carbol fuchsin stain. Heat was then applied beneath the slide until steam appeared. The slide was then left for 5 minutes whilst applying heat intermittently. The excess carbol fuchsin was washed off with tap water and excess water was drained off. The slide was then decolorized by covering with 20% H_2_SO_4_ and left for 2–5 minutes. It was subsequently counterstained with 3% methylene blue and left for 2 minutes after which it was washed with plenty of water, drained, and left to dry. The stained slide was then observed with the light microscope under oil immersion. Isolates of* M. ulcerans* confirmed by the described conventional laboratory methods were subjected to drug susceptibility testing to streptomycin (4 *μ*g/mL) and rifampicin (40 *μ*g/mL) by Canetti's proportion method for the determination of susceptibility of* Mycobacterium* to antibiotics [18]. For this method, briefly, dilutions of prepared standardized inocula were seeded onto drug-incorporated (tests) and drug-free (controls) Lowenstein-Jensen media. The proportions of resistant isolates were easily deduced by comparing counts from the controls and the tests.* M. ulcerans* isolates exhibiting growth on slants incorporated with the various test drugs were described as dual resistant isolates, whilst an isolate that grew only on one type of drug-incorporated slant exhibited monoresistance. Growth on either of the drug-incorporated media constituted monoresistance, whilst presumably susceptible isolates showed no growth on all drug-incorporated media.

### 2.2. Preparation of* M. ulcerans* Inoculum Suspension

*M. ulcerans* inoculum suspensions were prepared by picking two to three young and pure colonies of subcultured* M. ulcerans*, with a disposable 200 *μ*L capacity inoculating loop. To obtain a uniform sample suspension, the colonies were transferred to a sterile 7 mL bijou bottle containing 10–15 sterile glass beads (3 mg) and 500 *μ*L of sterile distilled water. Mixing was facilitated by intermittent vortexing for sixty seconds. Three millilitres (3 mL) of sterile distilled water was added to obtain a final volume of 4 mL. The receptacle was placed on flat surface to enable the sedimentation of the coarse particles in the suspension. The supernatant was carefully transferred to a sterile tube with a sterile disposable pipette [19, 20].

The turbidity of the* M. ulcerans* suspension was then adjusted to be equivalent to that of McFarland's standard 1 solution. This involved the addition of distilled water and matching with the standard solution equivalent to a concentration of 1 mg/mL of bacilli. Double dilutions of the standard suspension were done by adding 0.2 mL of suspension (10) to 1.8 mL of sterile distilled water to obtain a double dilution (10^−1^). This procedure was repeated to obtain a total of four dilutions of the bacteria suspension (10^−2^, 10^−3^, and 10^−4^).

### 2.3. Susceptibility Test and Interpretation of Results

For each test, 100 *μ*L of* M. ulcerans* inoculum dilutions was seeded onto Lowenstein-Jensen (L-J) media incorporated with streptomycin at a final drug dilution of 4 *μ*g/mL and rifampicin as 40 *μ*g/mL. Controls were set up as bacterial inocula on drug-free L-J media. A Ghanaian* M. ulcerans* strain (ATCC 970321-Ghana D19F9) was also set up as a control on both drug-free and drug-incorporated media. The L-J slopes were incubated at 32°C. Reading of the proportion test was done on 28th day and again on 42nd day. The growth was recorded as +++ for confluent growth and ++ for more than 100 colonies and for 1–99 colonies where the actual number of colonies was counted. Interpretation of all tests was based on the 42-day readings. For each isolate tested, the number of organisms resistant to each drug concentration was expressed as a percentage of the number of organisms growing on the drug-free slope. The selection of L-J slopes was made for estimating the growth on the drug-free and drug-containing media*. M. ulcerans* isolates expressing ≥1% growth on drug-containing media compared to that of drug-free media was considered resistant to the antibiotics rifampicin and streptomycin at test concentration of 40 *μ*g/mL and 4 *μ*g/mL, respectively.

## 3. Results

Seventy* M. ulcerans* isolates were tested for their susceptibility to streptomycin and rifampicin. In general, fourteen (14)* M. ulcerans* isolates exhibited resistance to either of the drugs in vitro (Table 1). Total resistance was expressed as the sum total of resistance to a specific drug individually or both. In this study, the highest resistance exhibited by the* M. ulcerans* isolates was to rifampicin (17.1%, 12/70) at 40 *μ*g/mL dilution, whilst that for streptomycin was 2.9% (2/70) at drug strength of 4 *μ*g/mL (Table 1). Majority of the* M. ulcerans* isolates exhibiting resistance were from the Asuboi Health Centre (10/70) in the Suhum-Kraboa-Coaltar district (Table 1). Out of 70 isolates tested, no isolate was found to be resistant to both antibiotics in vitro.

## 4. Discussion 

This study determined the susceptibility profiles of clinical* M. ulcerans* isolates to rifampicin and streptomycin in vitro by the Canetti agar proportion method [18]. A total of fourteen of* M. ulcerans* isolates exhibited in vitro resistance to either streptomycin or rifampicin; of these, twelve isolates exhibited resistance to rifampicin at a critical concentration of 40 *μ*g/mL, whilst resistance to streptomycin was exhibited by two of the* M. ulcerans* isolates tested. In vitro susceptibility and in vivo susceptibility to streptomycin and rifampicin have been reported by other BU studies [21, 22]. In most of these studies, the primary objective has been for drug discovery or improvement on existing regimen. Studies assessing susceptibility by clinical isolates is rare, though it is an important public health tool useful for effective disease surveillance, as it provides information on circulating resistant* M. ulcerans* within BU endemic communities [14].

Resistant MTB strains may simultaneously exhibit resistance to two or three anti-mycobacterial antibiotics which is generally attributable to multidrug exposure, a level of resistance rarely encountered in* M. ulcerans*. Initial challenging efforts at search for effective antibiotics warrant a more vigilant approach. In this study, there was no* M. ulcerans* isolate exhibiting dual resistance to rifampicin and streptomycin; this is not surprising as the low rate of* M. ulcerans* mutation has been reported in a study conducted by Jansson et al. [14] in Ghana. That notwithstanding, there is the need for continuous vigilance/monitoring on the possible emergence of resistant* M. ulcerans* strains in the BU endemic communities, especially where there could be cases of coinfections with* M. ulcerans* and MTB, which could aggravate the current condition. This study also reports on the current status of* M. ulcerans* isolates in two largely understudied BU endemic communities with a public health perspective on disease control. Studies in this direction are quite scanty, particularly on monitoring circulating resistant strains of* M. ulcerans*. In vitro determination of the susceptibility profiles of isolates obtained from clinical samples is an essential component for effective case management and public health [23, 24].

The present effective use of rifampicin and streptomycin with particularly large lesions (size > 15 cm) and also for the reported short healing durations ranging within 2–4 weeks raises expectations about the continuous use of antibiotics for effective management of the disease [8, 22]. In this study, two (2)* M. ulcerans* isolates showed resistance to streptomycin at a critical concentration of 4 *μ*g/mL. The results are comparable to others such as those conducted by Thangaraj et al., in 2000, where they also demonstrated the susceptibility of Ghanaian isolates to rifampicin in vitro and also in vivo in mice, by Dega et al., in 2000 [21, 22].

The activity of an antimicrobial is primarily to eliminate or inhibit growth of pathogens, a phenomenon which potentially facilitates the selection for drug resistance against therapeutic levels of recommended antibiotics [21, 25]. The phenomenon of drug resistance is one of the global health challenges in infectious conditions, including mycobacteriosis. Outcomes from several studies confirm this assertion [10, 22, 26, 27]. Investigating the susceptibility of an isolated infectious agent prior to the administration of an antibiotic is very important and is strongly recommended. This will help prevent improper exposure of infectious agents to inadequate levels of the drug, constituting an abuse that potentially leads to the selection of mutant isolates that could exhibit resistance to the recommended drug [28]. Mycobacteria therapy has had its fair share of multidrug resistance (MDR), with a lot of cases being reported particularly for* Mycobacterium tuberculosis* (MTB) [23, 29–31]. To date, however, there had been few reports of emergent resistant* M. ulcerans* isolates [8, 32]. This necessitates more studies into resistant* M. ulcerans* isolates. An effective drug formulation policy will depend on the information available on drug resistant profile of circulating* M. ulcerans* isolates. This will inform decision on antibiotic use, help review drug policy, and also introduce more effective drug combinations.

The current study was conducted in two communities recognised by the Ghana Health Service as BU endemic areas [12]. Even though the study may not be a representation of the overall status of BU drug resistance challenges in the whole country, it is important to know the situation at such endemic regions and to monitor possible emergence of resistant isolates. This monitoring activity is very necessary, especially with the current use of antibiotics for BU treatment. Generally, the prevalence of drug resistance among clinical* Mycobacterium ulcerans* isolates in Ghana has been reported to be low (0.9%) [32]. Meanwhile, there is no guarantee that the rate would continue to remain low. This is because drug resistance due to various factors do occur now and then and therefore require monitoring [22]. The type of resistance exhibited by the isolates used in the current was beyond the scope of this study and would be worth pursuing.

No isolate was found to be resistant to both streptomycin and rifampicin. This is a global as well as a regional phenomenon reported for most studies on drug susceptibility involving streptomycin and rifampicin and supports the high efficacy of streptomycin-rifampicin combination therapy [33]. There have also been reports of rifampicin resistance when used as monotherapy [23], similar to the current observation, since rifampicin and streptomycin have been administered for close to a decade, clearly indicating (i) relatively slow rate of mutation of the circulating* M. ulcerans* isolates and (ii) that the antibiotics are being adequately administered and, most importantly, effective compliance by the patients is limiting the potential of circulating isolates to mutate. As mentioned earlier, even though isolates used for the study were from patients with treatment failures and chronic wounds lasting longer than expected, most of the isolates did not exhibit resistance in vitro. Therefore, their unresponsiveness to treatment could be attributed to challenges associated with drug perfusion to the affected lesions as these chronic ulcers are also clearly necrotic. Also, outcome from the study cannot be projected to clinical* M. ulcerans* isolates obtained from normal healing wounds.

## 5. Conclusion

This study draws attention to the existence of* M. ulcerans* isolates exhibiting resistance to streptomycin and rifampicin in some BU endemic communities in Ghana. The low rate of mutation notwithstanding, there is still the need for periodic determination of DST of isolates as an effective monitoring and disease surveillance tool. This will enhance efforts at the control of Buruli ulcer disease, an important public health challenge.

## Figures and Tables

**Table 1:** Prevalence of resistant *M. ulcerans* isolates in two study districts.

Health centres	Resistant isolates of *M. ulcerans* *N* = 70		
	Rifampicin (40 *µ*g/mL) *n* (%)	Streptomycin (4 *µ*g/mL) *n* (%)	Total *n* (%)
Asuboi Health Centre	8 (11.4)	2 (2.9)	10 (14.3)
Paakro Health Centre	4 (5.7)	0 (0.0)	4 (5.7)
Total	12 (17.1)	2 (2.9)	14 (20.0)

*N* represents the total *M. ulcerans* isolates tested; *n* represents the number of resistant *M. ulcerans *isolates.

## Abbreviations

BU: Buruli ulcer
BUD: Buruli ulcer disease
*M. ulcerans*: * Mycobacterium ulcerans*
NMIMR: Noguchi Memorial Institute for Medical Research
GHS: Ghana Health Service
CFU: Colony forming unit
MDR: Multidrug resistance
WHO: World Health Organization
L-J: Lowenstein-Jensen
RIF: Rifampicin
STR: Streptomycin
NBUCP: National Buruli ulcer Control Programme
MTB: * Mycobacterium tuberculosis*
TB: Tuberculosis.
